# Liver injury and prolonged hospitalization as indicators of severity in patients with adenovirus infections

**DOI:** 10.1186/s12879-024-09324-x

**Published:** 2024-04-22

**Authors:** Shi Tang, Ru Qin, Dayong Zhang, Xiaoyan He, Chaowen Yu, Dapeng Chen, Xiaoqiang Li, Shan Liu

**Affiliations:** 1 Newborn Screening Center/Center for Clinical Molecular Laboratory Medicine, National Clinical Research Center for Child Health and Disorders, Ministry of Education Key Laboratory of Child Development and Disorders, Chongqing Key Laboratory of Pediatrics, Chongqing Medical University affiliated Children’s Hospital, 136 Zhongshan Er Road, Yuzhong District, 400014 Chongqing, China; 2 Clinical Laboratory of Chongqing, National Clinical Research Center for Child Health and Disorders, Ministry of Education Key Laboratory of Child Development and Disorders, Chongqing Key Laboratory of Pediatrics, Medical University affiliated Children’s Hospital, 400014 Chongqing, China

**Keywords:** Hospital stay, IFN-gamma, CD4-Positive T-Lymphocytes, Liver injury, Adenovirus infections

## Abstract

**Background:**

Adenovirus (ADV) is a prevalent infective virus in children, accounting for around 5–10% of all cases of acute respiratory illnesses and 4–15% of pneumonia cases in children younger than five years old. Without treatment, severe ADV pneumonia could result in fatality rates of over 50% in cases of emerging strains or disseminated disease. This study aims to uncover the relationship of clinical indicators with primary ADV infection severity, regarding duration of hospitalization and liver injury.

**Methods:**

In this retrospective study, we collected and analyzed the medical records of 1151 in-patients who met the inclusion and exclusion criteria. According to duration of hospitalization, all patients were divided into three groups. Then the difference and correlation of clinical indicators with ADV infection were analyzed, and the relationship among liver injury, immune cells and cytokines was evaluated.

**Results:**

The study revealed that patients with a duration of hospitalization exceeding 14 days had the highest percentage of abnormalities across most indicators. This was in contrast to the patients with a hospitalization duration of either less than or equal to 7 days or between 7 and 14 days. Furthermore, correlation analysis indicated that a longer duration of body temperature of ≥ 39°C, bilateral lung lobes infiltration detected by X ray, abnormal levels of AST, PaO2, and SPO2, and a lower age were all predictive of longer hospital stays. Furthermore, an elevated AST level and reduced liver synthesis capacity were related with a longer hospital stay and higher ADV copy number. Additionally, AST/ALT was correlated positively with IFN-γ level and IFN-γ level was only correlated positively with CD4^+^ T cells.

**Conclusions:**

The study provided a set of predicting indicators for longer duration of hospitalization, which responded for primary severe ADV infection, and elucidated the possible reason for prolonged duration of hospitalization attributing to liver injury via higher ADV copy number, IFN-γ and CD4^+^ T cells, which suggested the importance of IFN-γ level and liver function monitoring for the patients with primary severe ADV infection.

**Supplementary Information:**

The online version contains supplementary material available at 10.1186/s12879-024-09324-x.

## Background

Children are prone to be infected by viruses, which could arise everywhere in the human body, especially respiratory tract. Viral infection not only triggers severe diseases for example: pneumonia [1], asthma [2] and so forth, but is also secondary to immune dysfunction status, especially receiving chemotherapy for leukemia [3] and post-transplant [4]. But, the serotype, pathological and clinical characteristics and outcome are different between primary infection [5–6] and secondary infection [7]. Hence, it’s necessary to distinguish primary infection from secondary infection via patho-physiological characteristics.

Adenovirus (ADV) is a prevalent infective virus in children, accounting for around 5–10% of all cases of acute respiratory illnesses and 4–15% of pneumonia cases in children younger than five years old [8]. In addition, ADV infection can result in infrequent clinical symptoms such as conjunctivitis, hemorrhagic cystitis, hepatitis, hemorrhagic colitis, pancreatitis, nephritis, and meningoencephalitis [9–10]. Dissemination is more likely in patients with impaired immunity(for example organ transplant recipients, human immunodeficiency virus infection) [9–10]. Fatality rates for untreated severe ADV pneumonia with emergence of new strains [8] or disseminated disease may exceed 50% [9]. Given that the vast majority of primary cases are self-limited, the identification of clinical manifestations is important for administration and treatment the cases with ADV primary severe infection, effectively and inexpensively.

How to discriminate the severe viral infection is a systematic and comprehensive course. For severe acute respiratory syndrome coronavirus 2(SARS-CoV-2) infection, severe cases more frequently had dyspnea, lymphopenia, and hypoalbuminemia, with higher levels of alanine aminotransferase(ALT), lactate dehydrogenase, C-reactive protein, ferritin, and D-dimer as well as markedly higher levels of IL-2R, IL-6, IL-10, and TNF-α [11]. For Epstein-Barr virus(EBV) infection, aspartate aminotransferase(AST) level, number of CD8 + T cells, and CD62L expression on T cells were related to the severity of infectious mononucleosis [12]. For ADV infection, more data on co-infection were reported, for example human ADV type 7 and invasive pulmonary fungal co-infection [13], mycoplasma pneumoniae(MP) and ADV co-infection [14] and so forth. But, the changes of laboratory findings which are due to primary severe ADV single infection, are still unclear.

The length of hospital stay is usually regarded as an evaluation indicator for the effectiveness of intervention measures on diseases, for example enteral nutrition on severe acute pancreatitis [15], and as an indicator of relevant clinical outcomes in critically ill patients, for example ADV [13], COVID [16]. Duration of hospitalization as the outcome indicator for viral infection is feasible, its role and possible reason on severe ADV infection is worthy investigating further.

In the study, the difference of the clinical indicators related with severe infection was analyzed based on different duration of hospitalization. Then, the correlation of duration of hospitalization with indicators was calculated. Finally, the possible reason for longer duration of hospitalization was investigated. Our purpose is to evaluate the probability of duration of hospitalization as the outcome for severe ADV infection, uncover the predictors for duration of hospitalization and possible reason.

## Methods

### Study population

As a retrospective analysis, 1151 medical cases were collected, who were 1 months-15 years old patients and in-patients from January 1, 2018 to December 31, 2022 at Children´s Hospital of Chongqing Medical University, China.

### Inclusion and exclusion criteria

The inclusion criteria for the research group were as follows: (1) only in-patients were included; (2) ADV positive was detected by both two ways: ADV DNA by PCR and antigen by Immunofluorescence. Besides, children with any of the following factors were excluded: (1) congenital disease such as: congenital heart disease (2) inherited diseases, such as severe combined immunodeficiency (3) hospital acquired infection (4) chronic lung disease (5) chronic malnutrition (6) immunosuppression treatment.

### Clinical information collection

According to the above inclusion and exclusion criteria, 1151 patients were finally included for this study(Supplementary Fig. 1). The temperature, pulmonary imaging examination detected by x ray and ranked into three groups(no infiltration, infiltration only occurred in one side lung lobe and infiltration occurred in both lung lobes), general characteristics and laboratory examination(for example: white cell count, AST, immune cells, myocardial injury markers, blood gas analysis etc.) were obtained for retrospective analysis. The normal reference ranges of laboratory examination were listed in Supplementary Table 1.

### Statistical analysis

Categorical data was expressed as percentages and the Pearson chi-square test was used to compare the difference among groups. Normally distributed measurement data was expressed as means and standard deviations, while not normally distributed was represented by the first and third quartile. Comparisons of them were calculated by student’s t-tests. Ranked data was shown as the first and third quartile, which was compared by the chi-square test. Correlation analysis was also used to determine the related factors which could change the duration of hospitalization. A *p* value < 0.05 was considered statistically significant. All analysis were performed using SPSS version 17.0.

## Results

According to the inclusion and exclusion criteria, 1151 medical records were collected and analyzed. For general characteristics, ADV infection occurred in every season and the peak was in summer, the ratio of male/female was 1.868:1. Among the enrolled children, 471 cases(41.35%) were aged 2–5 years old, the following groups were respectively aged 1–2 years old(262 cases, 23%) and 3 months to 1 year old(238 cases, 20.9%). The infants under 3 months old were only 12 cases(1.05%)(Table 1).

**Table 1:** The characteristics of all patients

Parameter	No. / (Q1, Q3)	%
Total patients	1151	
**Year**	1151	
2018–2019	706	61.34
2020–2022	445	38.66
**Season**	1151	
Spring(Feb-Apr)	234	20.33
Summer(May-Jul)	521	45.26
Autumn(Aug-Oct)	139	12.08
Winter(Nov-Jan)	257	22.33
**Gender**	1150	
Male	749	65.13
Female	401	34.87
**Age**	1139	
< 3 m	12	1.05
3 m−1y	238	20.90
1−2y	262	23.00
2−5y	471	41.35
≥ 5y	156	13.70
**X-ray**	892	
Normal	253	28.36
^a^One side	123	13.79
^b^Both side	516	57.85
**High fever(≥ 39°C)**	1151	
Yes	680	59.08
No	471	40.92
**Duration of hospitalization(Days)**	(5, 10)	
**ADV copy number(×10** ^**6**^ **copies/ml)**	(7.91, 96.4)	

Note: a.One side, indicates that infiltration only occurs in the left lung lobe or right lung lobe; b.Both side, indicates that infiltration occurs in the both left and right lung lobe. Abbre. NO, number; Feb, February; Apr, April; Jul, July; Aug, August; Oct, October; Nov, November; Jan, January; m, month; y, year; S, Standard deviation; x,mean; Q1, quartile1;Q3, quartile 3

For the indicators relating to severity partly, ADV infected individuals with bilateral lung infiltration detected by X-ray was only 57.85%, and the individuals with body temperature ≥ 39°C was 59.08%. The duration of hospitalization, as a comprehensive indicator for severity, was from 1 day to 88 days, although the median of it was 9.24 days (Table 1). These data suggested that the severity of the children with ADV infection was different. Thus, subdivided data was needed to uncover the relationship of clinical characteristics with severe pediatric ADV infection.

Then, the duration of hospitalization as a consequence of ADV infection was categorised into three groups: group1 with duration of hospitalization ≤ 7 days, group 2 with duration of hospitalization 8–14 days and group 3 with duration of hospitalization > 14 days. And the difference of indicators related to severity were compared among three groups (Table 2). Compared to the group1, more cases were found in children aged < 2 years old in group 2(*p* < 0.001) and group3(*p* < 0.001). The percentage of patients with infiltration in two lung lobes was up from 39.64% in group 1 to 67.21% in group 2(*p* < 0.001) and 92.57% in group 3(*p* < 0.001), and body temperature ≥ 39°C was up from 45.62% in group 1 to 73.35% in group 2(*p* < 0.001) and 84.11% in group 3(*p* < 0.001). For more indicators, compared to group1, the significant difference were observed in group3, on abnormal percentage of RBC count(*p* = 0.022), hemoglobin(Hb) concentration(*p* = 0.013), white blood cell(WBC) count(*p* = 0.013), neutrophil ratio(*p* < 0.001), lymphocyte ratio(*p* < 0.001), Aspartate aminotransferase(AST) (*p* < 0.001), glutamic-pyruvic transaminase(ALT)(*p* < 0.001), Albumin(*p* < 0.001), troponin(TnI)(*p* < 0.001), B-type brain natriuretic peptide(BNP)(*p* = 0.030), arterial oxygen partial pressure(PaO2)(*p* = 0.037) and oxygen saturation of blood(SPO2)(*p* = 0.017); but the significant difference was observed in group2, only on neutrophil ratio(*p* = 0.045), AST(*p* < 0.001) and Albumin(*p* < 0.001). The data proved that longer duration of hospitalization(especially > 14 days) was accompanied with more abnormal indicators related to severity.

**Table 2:** The difference of clinical characteristics based on different duration of hospitalization

Parameter	≤ 7 days	8–14 days		> 14 days		p^1^ value
	No.(%)	No.(%)	p^2^ value	No.(%)	p^3^ value	
Total No.	651	349		151		
**Age**	644	345	< 0.001	150	< 0.001	< 0.001
< 3 m	3(0.46)	6(1.74)		3(2.00)		
3 m−1y	72(11.18)	102(29.56)		64(42.67)		
1−2y	125(19.41)	93(26.96)		44(29.33)		
2−5y	327(50.78)	111(32.17)		33(22.00)		
≥ 5y	117(18.17)	33(9.57)		6(4.00)		
**X-ray**	439	305	< 0.001	148	< 0.001	< 0.001
Normal	201(45.78)	47(15.41)		5(3.38)		
^a^One side	64(14.58)	53(17.38)		6(4.05)		
^b^Both side	174(39.64)	205(67.21)		137(92.57)		
**High fever(≥ 39°C)**	651	349	< 0.001	151	< 0.001	< 0.001
	297(45.62)	256(73.35)		127(84.11)		
**Blood system**						
Abnormal RBC count	150(24.47)	98(28.74)	0.423	49(35.58)	0.022	0.064
Abnormal Hb	174(28.20)	118(34.50)	0.309	57(44.88)	0.013	0.041
Abnormal WBC count	187(30.21)	114(33.33)	0.648	57(47.24)	0.013	0.029
Abnormal Neutrophil(%)	214(34.63)	168(49.12)	0.045	83(65.87)	< 0.001	< 0.001
Abnormal lymphocyte(%)	150(24.35)	94(27.49)	0.626	63(50.50)	< 0.001	< 0.001
Abnormal CRP	571(98.45)	332(98.52)	0.561	122(97.60)	1	0.816
**Liver function**						
Abnormal AST	115(18.91)	133(39.12)	< 0.001	81(54.36)	< 0.001	< 0.001
Abnormal ALT	58(9.54)	37(10.88)	0.509	35(23.49)	< 0.001	< 0.001
**Kidney function**						
Abnormal Creatinine	2(0.33)	4(1.18)	0.113	1(0.67)	0.553	0.288
Abnormal Urea	50(8.20)	34(10.00)	0.348	15(10.00)	0.479	0.583
Abnormal Albumin	126(20.59)	112(32.75)	< 0.001	79(52.67)	< 0.001	< 0.001
**Myocardial injury**						
Abnormal TnI	14(2.35)	19(5.78)	0.149	28(24.56)	< 0.001	< 0.001
Abnormal CKMB	49(8.24)	30(9.09)	0.800	16(14.04)	0.175	0.328
Abnormal BNP	6(1.76)	5(2.87)	0.651	6(8.96)	0.030	0.040
**Blood gas analysis**						
Abnormal PH	66(47.48)	49(38.28)	0.198	50(46.73)	1.000	0.334
Abnormal PaO_2_	102(73.38)	88(68.75)	0.533	91(85.05)	0.037	0.023
Abnormal SPO_2_	21(15.11)	17(13.28)	0.684	31(28.97)	0.017	0.007

Note: a.One side, indicates that infiltration only occurs in the left lung lobe or right lung lobe; b.Both side, indicates that infiltration occurs in the both left and right lung lobe; *p*1, comparison among three groups. *p*2 and *p*3, compared to the group with duration of hospitalization ≤ 7 days. Abbre. NO., number; m, month; y, year; RBC, red blood cell; Hb, hemoglobin; WBC, white blood cell; CRP, C-reactive protein; AST, Aspartate aminotransferase; ALT, glutamic-pyruvic transaminase; TnI, troponin; CKMB, creatine kinase isoenzyme; BNP, B-type brain natriuretic peptide; PaO_2_, arterial oxygen partial pressure; SPO_2_, oxygen saturation of blood. *p* value was calculated by Pearson chi-square test

Moreover, the factors which showed a statistically significant difference among different groups were analyzed to explore the correlation with duration of hospitalization. As shown in Table 3, we found that infiltration(*r* = 0.520), days with a body temperature ≥ 39°C(*r* = 0.413), abnormal AST levels(*r* = 0.303), PaO_2_(*r* = 0.332) and SPO_2_ (*r* = 0.340) had a positive correlation with duration of hospitalization, while age had a negative correlation, which demonstrated that wider infiltration, longer time with a body temperature ≥ 39°C, abnormal AST levels, PaO2 and SPO_2_, and a lower age predicted longer duration of hospitalization for the patients with ADV infection.

**Table 3:** The correlation of hospital time with other parameters

Parameter	(Q1,Q3)/${\bf{\bar x}}\, \pm \,{\bf{s}}$	*p* value	r
Age	(1.080,4.000)	< 0.001	−0.352
^*^Infiltration	(1,3)	< 0.001	0.520
High fever(≥ 39°C) Time	2.140±2.930	< 0.001	0.413
RBC count	4.330±0.520	< 0.001	−0.149
Hb	112.190±13.305	< 0.001	−0.241
WBC count	9.880±5.673	< 0.001	0.091
Neutrophil(%)	(0.420,0.700)	< 0.001	0.282
Lymphocyte(%)	(0.260,0.518)	< 0.001	0.201
AST	(30.400,56.600)	< 0.001	0.303
ALT	(12.000,29.3000)	< 0.001	0.124
Albumin	39.89±12.405	< 0.001	−0.149
TnI	(0.000,0.010)	< 0.001	0.142
BNP	(5.695,18.855)	0.363	0.027
PaO_2_	(61.000,103.000)	< 0.001	0.332
SPO_2_	(0.910,0.980)	< 0.001	0.340

Note: Non normally distributed measurement data was shown as(Q1,Q3); normally distributed measurement data was shown as‾x ± s. * ranked data, 1 indicates no infiltration, 2 indicates that infiltration only occurs in the left lung lobe or right lung lobe, 3 indicates that infiltration occurs in the both left and right lung lobe. Abbre. RBC, red blood cell; Hb, hemoglobin; WBC, white blood cell; AST, Aspartate aminotransferase; ALT, glutamic-pyruvic transaminase; TnI, troponin; BNP, B-type brain natriuretic peptide; PaO_2_, arterial oxygen partial pressure; SPO_2_, oxygen saturation of blood. *p* for normally distributed measurement data was calculated by Pearson; *p* for not normally distributed measurement data and ranked data were calculated by Spearman

**Fig. 1:**
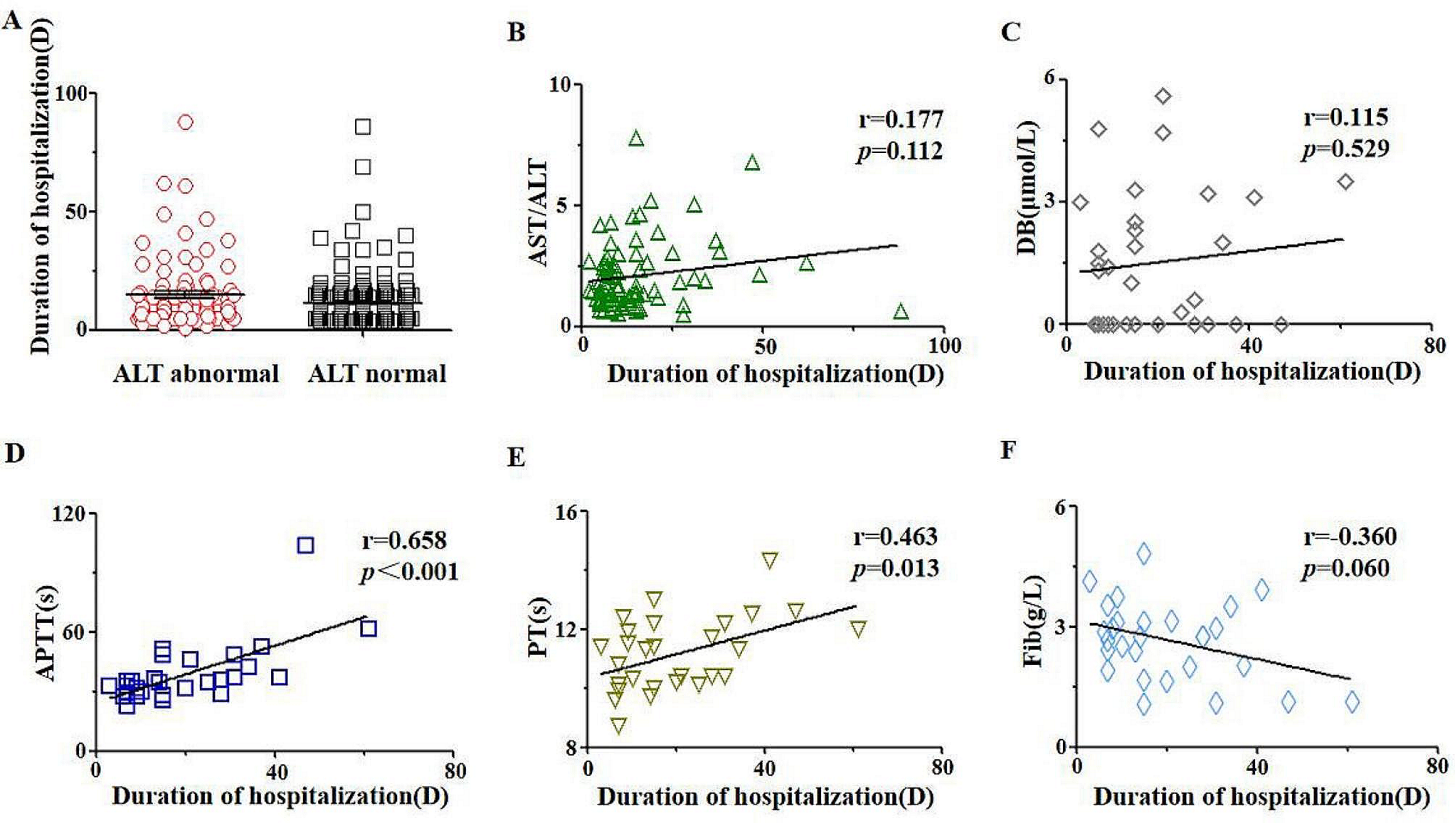
The correlation of duration of hospitalization with liver function (**A**)The comparison of duration of hospitalization between the patients with abnormal level of ALT (*n* = 94) and with normal level of ALT (*n* = 94 vs. *n* = 234, *p* = 0.206). For the patients with abnormal level of ALT, the evaluation was performed on the relationship of duration of hospitalization with liver function via the indicators including: AST/ALT(**B**), DB(**C**), APTT(**D**), PT(**E**) and Fib(**F**). Note: AST/ALT, the ratio of the value of AST and ALT. Abbre.: AST: Aspartate aminotransferase; ALT: glutamic-pyruvic transaminase; DB: direct bilirubin; APTT: activated partial thromboplastin time; PT: Prothrombin time; Fib: fibrinogen; D: days. r was calculated by spearman correlation coefficient

According to Divya [17], ADV infection can cause liver damage and we also demonstrated that AST/ALT was correlated positively with the duration of hospitalization (Fig. 1B). Thus, the role of severe liver damage on duration of hospitalization was further investigated. Based on abnormal AST level(*n* = 328), the patients with abnormal ALT level(*n* = 94) had longer duration of hospitalization(average time = 14.77 days) than the patients with normal ALT level(*n* = 234, average time = 11.32 days)(Fig. 1A). Excluding the the abnormal dead patients and the patients with low level ALT, 83 patients with high levels of ALT and AST were analyzed for additional indicators related to liver damage. Among 83 patients, direct bilirubin(DB), activated partial thromboplastin time(APTT), ProthrombinTime(PT) and Fibrinogen(Fib) were detected in 32 patients, and we found that APTT (Fig. 1D, *r* = 0.658, *p* < 0.001), PT (Fig. 1E, *r* = 0.463, *p* = 0.013) were correlated significantly positively with duration of hospitalization, and the correlation is slight on DB level (Fig. 1C, *r* = 0.115, *p* = 0.529), Fib concentration (Fig. 1F, *r*=-0.360, *p* = 0.060). The data declared that severe liver damage suggested the longer duration of hospitalization for patients with ADV infection.

**Fig. 2:**
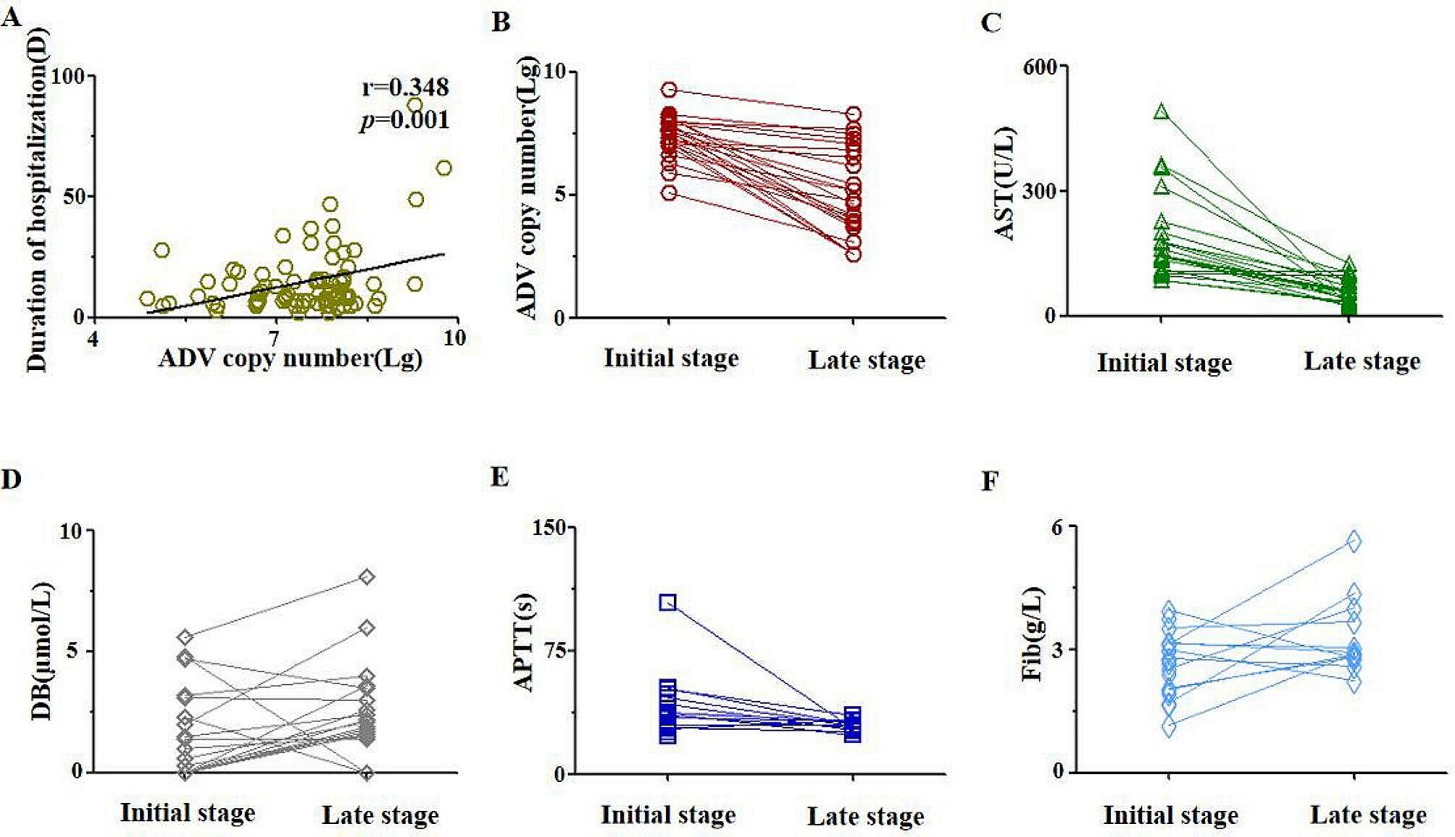
The relationship of ADV copy number with liver function (**A**)The correlation analysis of ADV copy number(Lg) with duration of hospitalization. (**B**)The comparison of ADV copy number(lg) between the initial and late stage of disease. The comparison of liver function related factors including: AST(**C**), DB(**D**), APTT(**E**), Fib(**F**) between the initial and late stage of disease Note: Initial stage, the first detection of ADV DNA using PCR during hospitalization; Late stage, the last detection of ADV DNA using PCR during hospitalization. Abbre.: AST: Aspartate aminotransferase; DB: direct bilirubin; APTT: activated partial thromboplastin time; Fib: fibrinogen; D: days; ADV: Adenovirus; Lg: logarithmic value of 10. r was calculated by spearman correlation coefficient

To elucidate the possible cause for liver damage by ADV, we firstly confirmed the effect of single ADV infection on liver damage, because of multiple infection with ADV occurring in children frequently. We analyzed the correlation of ADV copy number with duration of hospitalization. Interestingly, the result significantly showed that the two of them had a positive correlation (Fig. 2A, *r* = 0.348, *p* = 0.001). Then, multiple infection was analyzed. We found that there was no statistical significance between the single infection group(*n* = 62) and multiple infection group(*n* = 21) on duration of hospitalization and even AST/ALT ratio(Supplementary Fig. 2), which might suggest that ADV had a major role on the liver damage, but not other pathogenic organisms.

Next, 24 patients with single ADV infection were compared the data reflecting liver function in initial and late stage of illness process. In late stage, decreased ADV copy number(*p* < 0.001) (Fig. 2B), decreased AST level(*p* < 0.001) (Fig. 2C), increased DB level(*p* = 0.055) (Fig. 2D), shortened APTT(*p* = 0.039) (Fig. 2E) and increased Fib level(*p* = 0.101) (Fig. 2F) were observed. The evidence uncovered that severe liver damage was related with viral copy number in the patients with ADV infection.

**Fig. 3:**
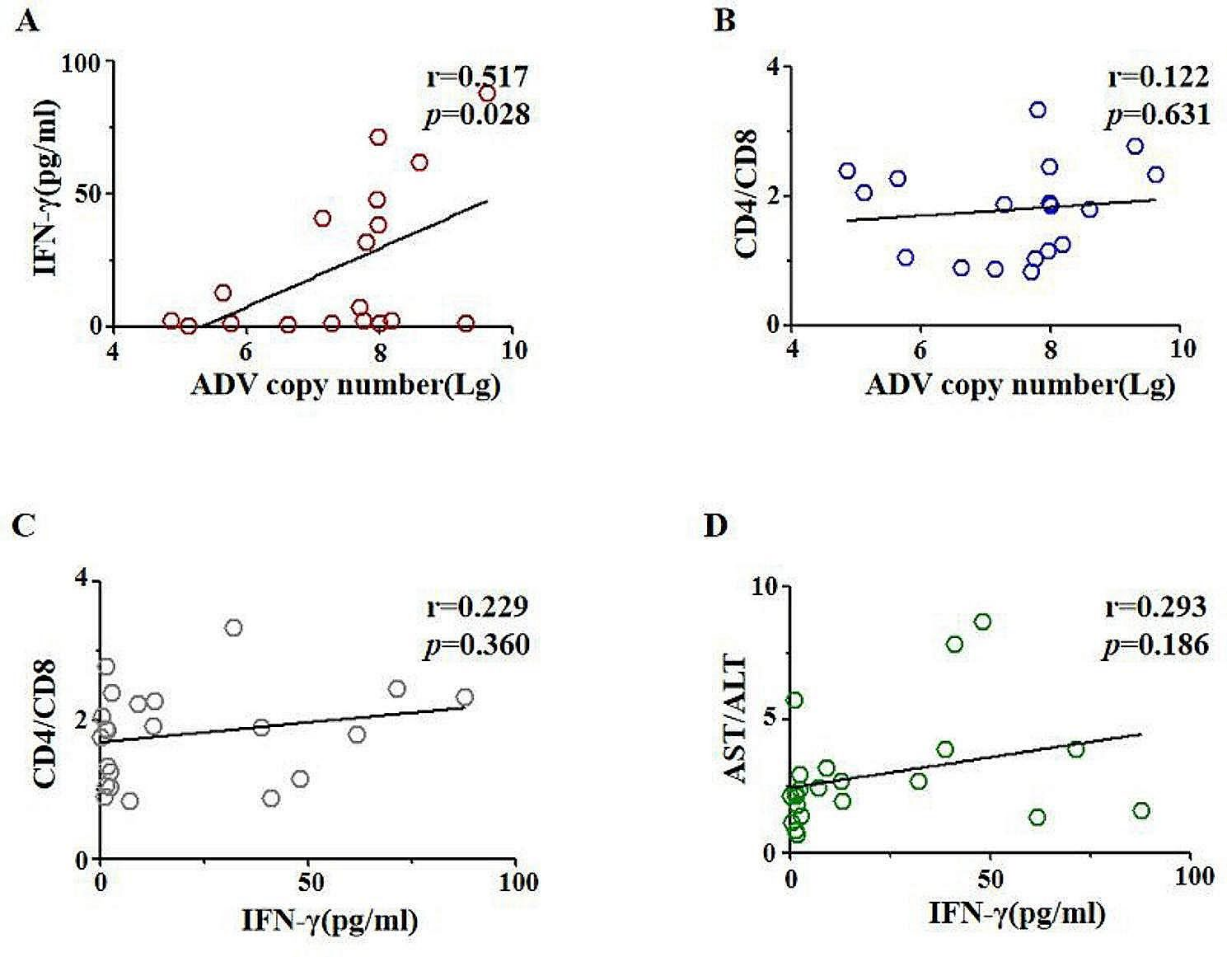
The relevance of liver damage with IFN-γ The correlation analysis of Adv copy number(Lg) with IFN-γ(**A**) and CD3 + CD4 + T cell/CD3 + CD8 + T cells(**B**). The correlation analysis of IFN-γ with CD3 + CD4 + T cell/CD3 + CD8 + T cells(**C**) and AST/ALT(**D**). Note: CD4/CD8, the ratio of CD3 + CD4 + T cells and CD3 + CD8 + T cells. Abbre.: IFN-γ: Interferon-γ; ADV: Adenovirus; Lg: logarithmic value of 10. r was calculated by spearman correlation coefficient

Further, we tried to analyze the role of immune-mediated liver damage [18] on the progress of ADV infection. Different cytokines and immune cells were investigated. AST and ALT level were related positively with multiple cytokines, especially IFN-γ(Fig. 3D, *r* = 0.293 *p* = 0.186), and was correlated positively with CD4 + T cells(Supplementary Fig. 5A). Moreover, the IFN-γ level positively related with CD4 + T cells/CD8 + T cells ratio (Fig. 3C, *r* = 0.229 *p* = 0.360). These data indicated that IFN-γ and CD4 + T cells were important for immune-mediated liver damage in children with ADV infection.

Additionally, ADV copy number had a strong correlation with the level of IFN-γ (Fig. 3A, *r* = 0.517 *p* = 0.028). Although both CD4 + T cells and CD8 + T cells had a negative correlation with ADV copy number, the ratio of CD4/CD8 had a slightly positive correlation with ADV copy number (Fig. 3B, *r* = 0.122 *p* = 0.631), which suggested that severe liver damage caused by ADV infection might correlate with higher IFN-γ level and CD4 + T cells.

## Discussion

In the study, 1151 hospitalized cases who were selected for analysis could be defined as the median to severe ADV infection. The infection was confirmed through detection of ADV using both immunofluorescence at the protein level and PCR at the DNA level. According the detective methods, the patients only with higher ADV copy number(Q1 = 7.91 × 10^6^copies/ml, Q3 = 9.64 × 10^7^copies/ml) were included. Therefore, the population had more severity than previous reporter [10], for example the longer average duration of hospitalization. Besides, patients with congenital diseases and inherited diseases, such as congenital heart disease and severe combined immunodeficiency, were excluded from the study. Thus, all 1151 patients met the need of inclusion and exclusion criteria for primary median/severe ADV infection.

Based on duration of hospitalization, the population were dived into three groups. The average duration of hospitalization was about 14 days for the patients with abnormal AST and ALT (Fig. 1A), and the duration of hospitalization for self-limiting viral infection was 7 days. So, the duration of hospitalization was dived into three groups: ≤7 days, 8–14 days and > 14 days. Obviously, compared to the patients with duration of hospitalization ≤ 7 days, the patients with duration of hospitalization > 14 days had higher percentage of abnormal indicators related with severity (Table 2) and were correlated to the abnormal degree (Table 3). These data implied that duration of hospitalization > 14 days, as a comprehensive indicator related with all path-physiological status of the body, could reflect the severity and be regarded as the outcome indicator for the children with severe ADV infection.

Although wider infiltration, longer periods with a body temperature of ≥ 39°C, abnormal AST level, PaO2, SPO2, and lower age were found to predict longer hospitalization duration (Table 3), acute liver damage in children was a rapidly evolving catastrophic clinical condition that resulted in high mortality/morbidity [17] and AST indicated dysfunction in single system. Thus, only liver damage was analyzed further. More data was needed for the role of other indicators on duration of hospitalization.

As known to us, viral liver damage increased AST, ALT [19–20] and LDH levels, reduced the total bilirubin level and decreased liver synthesis such as antithrombin III, partial thromboplastin time and coagulation factors II, V and VII [20]. In our study, accompanied with decreased ADV copy number, decreased AST, increased DB and Fib, and shortened APTT (Fig. 2) were observed and these changed indicator might be due to recovery synthesis function partly [21]. Meanwhile, the duration of hospitalization suggested the higher copy number of ADV (Fig. 2A), and the increased AST, APTT and PT and decreased Fib were related with longer duration of hospitalization. These data implied that liver damage caused by ADV infection could predict the longer duration of hospitalization.

Atasheva and colleagues [22] found that low dose adenovirus exposure in human immune cells triggers a pro-inflammatory response within 72 h. This response involves a specific set of cytokines, such as IL4, IL6, TNFα, and IFN-γ. In our study, except for TNFα, other cytokines exhibited higher expression trend accompanied with higher ADV copy number, but the significant difference was only observed on IFN-γ. Fu Y et al. found that cytokine level depended on infected ADV subtype in mouse BALF [23]. Thus, whether mixed subtype narrowed the difference of cytokines caused by ADV infection, needed more specimens to identify.

For the patients with abnormal AST and ALT, on the hand, CD4 + T cells(average ratio:36.67%) and CD8 + T cells(average ratio:24.65%) were decreased, which might be explained by that elevated exhaustion levels T cells was derived from immune dysregulation caused by severe infection [24]; On the other hand, NK cells(average ratio:7.87%) and CD19 + cells(average ratio:27.11%) were increased slightly caused by ADV infection(Supplementary Fig. 4). But, the ratio of all cells was still in normal reference range [25](Supplementary Fig. 4F). Considering that the absolute number of CD4 + T cells exceeds that of NK cells, we deduced that IFN-γ was mainly produced by CD4 + T cells. As shown in Supplementary Fig. 5, the speculation was demonstrated by only the number of CD4 + T cells positively relating to the level of IFN-γ expression.

After exposing to viruses, IFN-γ-producing NK cells was positively associated with IFN-γ-producing CD4 + T cells [26]. Additionaly, CD4 + T cells enhanced production of IFN-γ by NK cells [27] and NK cells positively regulated CD8 + T cells via secreting IFN-γ [28]. What’s more, increased IFN-γ expression in CD4 + T and CD8 + T cells was related with decreased bilirubin production and exacerbated liver immunopathology [29–30], which might explained the cause of the positive relationship of AST/ALT with IFN-γ level partly.

In conclusion, patients with ADV infection hospitalized for more than 14 days displayed a higher percentage of severity-associated abnormal indicators. Additionally, liver damage was linked to longer hospital stays which was related with IFN-γ and CD4 + T cells caused by ADV infection. These data indicated the importance of IFN-γ level and liver function monitoring for the patients with primary severe ADV infection.

### Electronic supplementary material

Below is the link to the electronic supplementary material.

Supplementary Material 1

Supplementary Material 2

Supplementary Material 3

Supplementary Material 4

Supplementary Material 5

Supplementary Material 6

## Data Availability

The datasets used and/or analyzed during the current study are available from the corresponding author on reasonable request.

## Abbreviations

ˉx: Mean
ADV: Adenovirus
ALT: Glutamic-pyruvic transaminase
Apr: April
APTT: Activated partial thromboplastin time
AST: Aspartate aminotransferase
Aug: August
BNP: B-type brain natriuretic peptide
CKMB: Creatine kinase isoenzyme
CRP: C-reactive protein
D: Days
DB: Direct bilirubin
Feb: February
Fib: Fibrinogen
Hb: Hemoglobin
IFN-γ: Interferon-γ
IL−10: Interleukin−10
IL−2: Interleukin−2
IL−4: Interleukin−4
IL−6: Interleukin−6
Jan: January
Jul: July
Lg: Logarithmic
m: Month
No: Number
Nov: November
Oct: October
PaO_2_: Arterial oxygen partial pressure
PCR: Polymerase chain reaction
PT: Prothrombin time
Q1: Quartile1
Q3: Quartile3
RBC: Red blood cell
s: Standard deviation
SPO2: Oxygen saturation of blood
TNF-α: Interferon-α
TnI: Troponin
WBC: White blood cell
y: Year
