# Supplementary material for: Liver injury and prolonged hospitalization as indicators of severity in patients with adenovirus infections
Source: BMC Infect Dis. 2024 Apr 22;24:430. doi: 10.1186/s12879-024-09324-x (PMC11036557; doi:10.1186/s12879-024-09324-x)
Supplement: Supplementary file 1 — Supplementary Material 1 [file 12879_2024_9324_MOESM1_ESM.pdf]

Supplementary Table 1 The normal reference ranges of laboratory examination

Parameter	Female	Male	Age range
**Blood system**			
RBC(×10⁶/μL)	4.3-6.4	4.3-6.4	0-24 h
	4.0-6.8	4.0-6.8	25-144 h
	3.7-6.1	3.7-6.1	7-14 d
	3.2-5.4	3.2-5.4	15-28 d
	3.3-5.2	3.3-5.2	29-180 d
	4.0-5.5	4.0-5.5	6-12 m
	4.1-5.5	4.1-5.5	1-＜6 y
	4.2-5.7	4.2-5.7	6-＜13 y
	4.1-5.4	4.4-5.8	13-＜16 y
Hb(g/L)	152-235	152-235	0-24 h
	150-240	150-240	25-144 h
	127-187	127-187	7-14 d
	103-185	103-185	15-28 d
	97-183	97-183	29-180 d
	97-141	97-141	6-12 m
	107-141	107-141	1-＜2 y
	112-149	112-149	2-＜6 y
	118-156	118-156	6-＜13 y
	110-154	120-170	13-＜16y
WBC(×10³/μL)	5.0-30.0	5.0-30.0	0-72 h
	5.0-21.0	5.0-21.0	4-7 d
	5.0-20.0	5.0-20.0	8-14 d
	5.0-19.5	5.0-19.5	15-28 d
	4.3-14.2	4.3-14.2	29-180 d
	4.8-14.6	4.8-14.6	6-12 m
	5.1-14.1	5.1-14.1	1-＜2 y
	4.4-11.9	4.4-11.9	2-＜5 y
	4.3-11.3	4.3-11.3	6-＜13 y
	4.1-11.0	4.1-11.0	13-＜19 y
Neutrophil(%)	0-88	0-88	0-24 h
	0-71	0-71	25-144 h
	0-66	0-66	7-14 d
	5-60	5-60	15-28 d
	7-56	7-56	29-180 d
	9-57	9-57	6-12 m
	13-55	13-55	1-＜2 y
	22-65	22-65	2-＜6 y
	31-70	31-70	6-＜13 y
	37-77	37-77	13-＜19 y
Lymphocyte(%)	0-40	0-40	0-144 h

	0-60	0-60	7-14 d
	20-60	20-60	15-28 d
	26-83	26-83	29-180 d
	31-81	31-81	6-12 m
	33-77	33-77	1-＜2 y
	23-69	23-69	2-＜6 y
	23-59	23-59	6-＜13 y
	17-54	17-54	13-＜19 y
CRP(mg/L)	＜8	＜8	＜16 y
**Liver function**			
	5.0-101.0	5.0-101.0	0-27 d
	21.0-80.0	21.0-80.0	28d-＜1 y
AST(U/L)	22.0-59.0	22.0-59.0	1-＜2 y
	14.0-44.9	14.0-44.9	2-＜13 y
	10.0-31.0	12.0-37.0	13-＜18 y
	5.0-71.0	5.0-71.0	0-27 d
	8.0-71.0	8.0-71.0	28d-＜1 y
ALT(U/L)	8.0-45.9	8.0-45.9	1-＜2 y
	7.0-44.8	7.0-44.8	2-＜13 y
	6.0-44.9	7.0-45.4	13-＜18 y
**Kidney function**			
	28.0-44.0	28.0-44.0	0-27 d
Albumin(g/L)	28.0-50.0	28.0-50.0	28 d-＜6 m
	37.9-54.0	37.9-54.0	6 m-＜13 y
	39.9-56.0	42.0-56.0	13-＜18 y
	13.0-82.0	13.0-82.0	0-＜3 m
	13.0-56.5	13.0-56.5	3 m-＜2 y
Creatinine(μmol/L)	14.6-56.5	14.6-56.5	2-＜4 y
	19.0-52.5	19.0-52.5	4-＜6 y
	27.0-66.0	27.0-66.0	6-＜13 y
	29.2-88.6	33.0-93.0	13-＜16 y
	0.71-5.72	0.71-5.72	0-＜3 m
	0.8-6.1	0.8-6.1	3-＜6 m
Urea(mmol/L)	1.1-6.1	1.1-6.1	6-12 m
	1.3-6.7	1.3-6.7	1-＜2 y
	1.3-6.5	1.3-7.0	2-＜4 y
	2.1-7.1	2.1-7.1	4-＜18 y
**Myocardial injury**			
TnI(μg/L)	<0.06	<0.06	＜16 y
CKMB(μg/L)	0.21-5.0	0.21-5.0	＜16 y
BNP(pg/L)	<100	<100	＜16 y
**Blood gas analysis**			
PH	7.35-7.45	7.35-7.45	＜16 y
PO_2(mmHg)	80-100	80-100	＜16 y

SO_2	0.9-1	0.9-1	＜16 y

Note: RBC, red blood cell; Hb, hemoglobin; WBC, white blood cell; CRP, C-reactive protein; AST, glutamic oxaloacetic transaminase; ALT, glutamic-pyruvic transaminase; TnI, troponin;
CKMB, creatine kinase isoenzyme; BNP, B-type brain natriuretic peptide; PaO_2, arterial oxygen partial pressure; SO_2, oxygen saturation of blood; h, hour; d, day; m, month; y, year.
