# Supplementary figures and images for: Liver injury and prolonged hospitalization as indicators of severity in patients with adenovirus infections
Source: BMC Infect Dis. 2024 Apr 22;24:430. doi: 10.1186/s12879-024-09324-x (PMC11036557; doi:10.1186/s12879-024-09324-x)

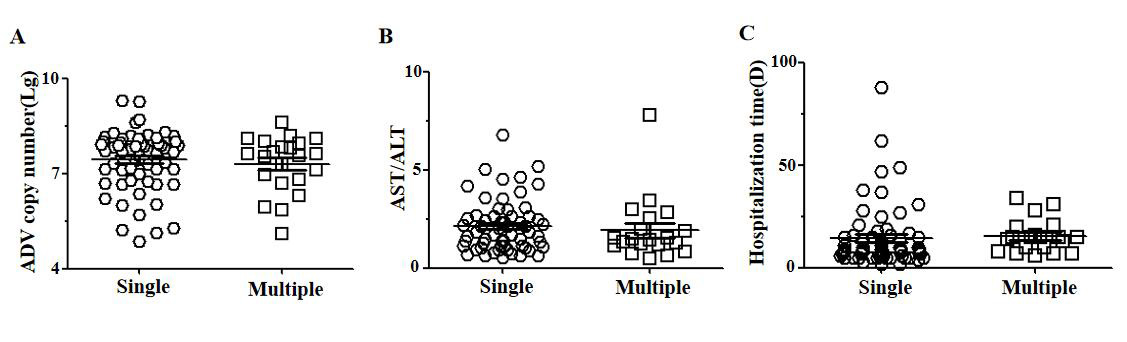

Supplement: Supplementary file 2 — Supplementary Material 2 [file 12879_2024_9324_MOESM2_ESM.jpg]

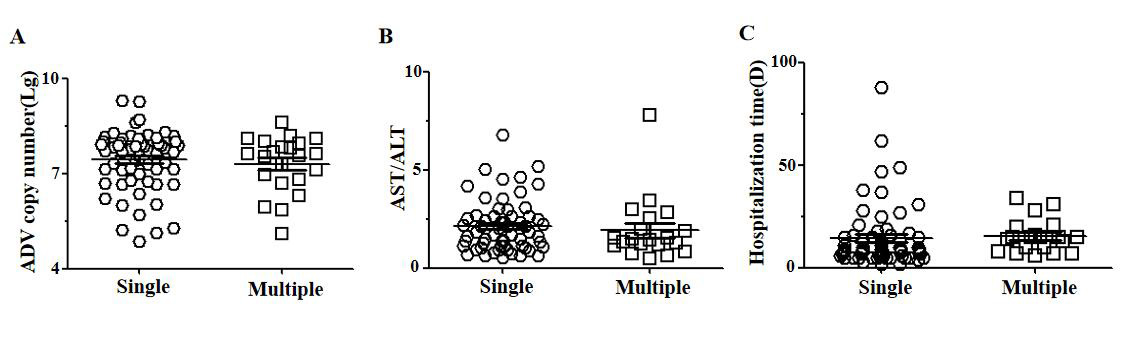

Supplement: Supplementary file 3 — Supplementary Material 3 [file 12879_2024_9324_MOESM3_ESM.jpg]

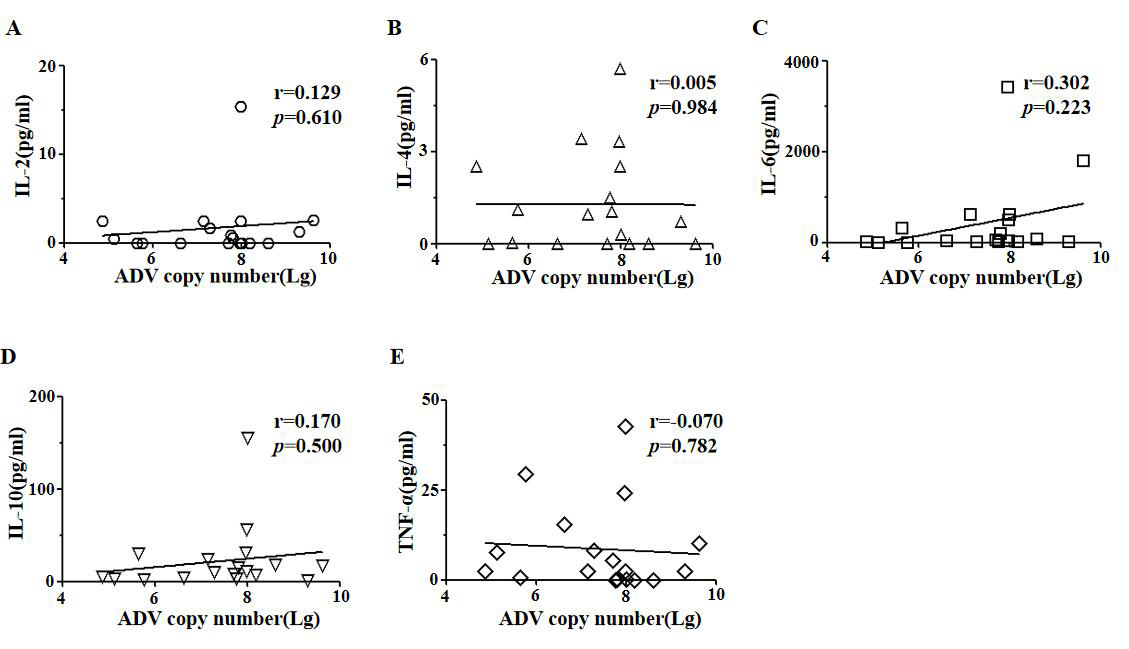

Supplement: Supplementary file 4 — Supplementary Material 4 [file 12879_2024_9324_MOESM4_ESM.jpg]

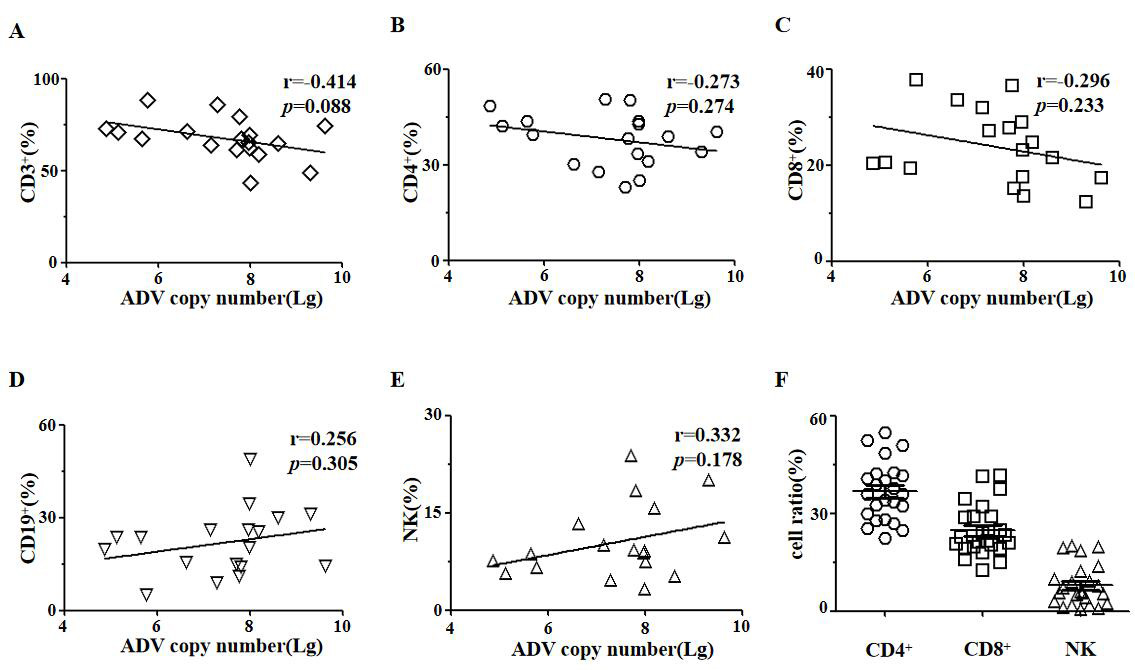

Supplement: Supplementary file 5 — Supplementary Material 5 [file 12879_2024_9324_MOESM5_ESM.jpg]

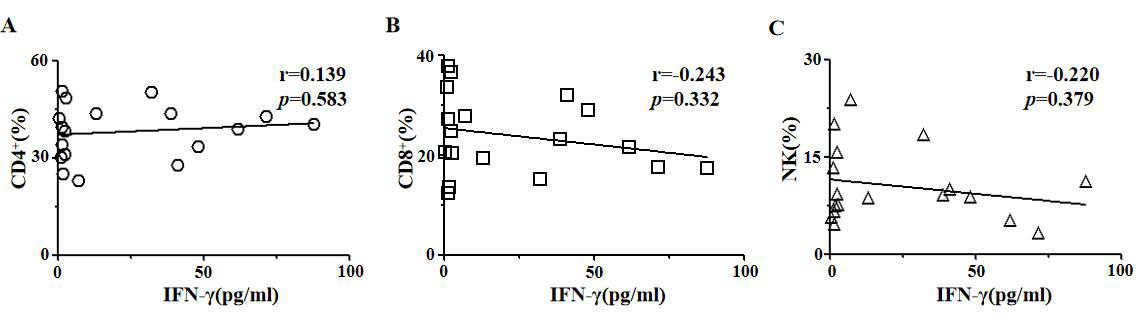

Supplement: Supplementary file 6 — Supplementary Material 6 [file 12879_2024_9324_MOESM6_ESM.jpg]
